# Transcriptional Effects of Glucocorticoid Receptors in the Dentate Gyrus Increase Anxiety-Related Behaviors

**DOI:** 10.1371/journal.pone.0007704

**Published:** 2009-11-02

**Authors:** Nadège Sarrazin, Francesco Di Blasi, Valérie Roullot-Lacarrière, Françoise Rougé-Pont, Anne Le Roux, Pierre Costet, Jean-Michel Revest, Pier Vincenzo Piazza

**Affiliations:** 1 Pathophysiology of Addiction group, Neurocenter Magendie, INSERM U862, Bordeaux, France; 2 Université de Bordeaux, Bordeaux, France; 3 Istituto di Biomedicina e di Immunologia Molecolare, CNR, Palermo, Italy; 4 Transgenesis Laboratory, Université de Bordeaux, Bordeaux, France; University of Parma, Italy

## Abstract

The Glucocorticoid Receptor (GR) is a transcription factor ubiquitously expressed in the brain. Activation of brain GRs by high levels of glucocorticoid (GC) hormones modifies a large variety of physiological and pathological-related behaviors. Unfortunately the specific cellular targets of GR-mediated behavioral effects of GC are still largely unknown. To address this issue, we generated a mutated form of the GR called ΔGR. ΔGR is a constitutively transcriptionally active form of the GR that is localized in the nuclei and activates transcription without binding to glucocorticoids. Using the tetracycline-regulated system (Tet-OFF), we developed an inducible transgenic approach that allows the expression of the ΔGR in specific brain areas. We focused our study on a mouse line that expressed ΔGR almost selectively in the glutamatergic neurons of the dentate gyrus (DG) of the hippocampus. This restricted expression of the ΔGR increased anxiety-related behaviors without affecting other behaviors that could indirectly influence performance in anxiety-related tests. This behavioral phenotype was also associated with an up-regulation of the MAPK signaling pathway and Egr-1 protein in the DG. These findings identify glutamatergic neurons in the DG as one of the cellular substrate of stress-related pathologies.

## Introduction

Glucocorticoid hormones (GC) are the end product of the activation of the hypothalamus-pituitary-adrenal (HPA) axis. The secretion of these hormones increases during the active phase of the circadian cycle and in response to stress [1], [2]. Glucocorticoids through their action on the brain have large effects on adaptive behaviors and are involved in the pathophysiology of several stress-related disorders such as drug abuse, depression and anxiety [2]–[5], [5]–[9].

Most of the behavioral and stress-related effects of glucocorticoids depend on the activation of Glucocorticoid Receptors (GR). GRs are hormone-activated transcription factors [10] that upon binding to glucocorticoids, translocate to the nucleus where they modify the expression of target genes through many different molecular mechanisms [11].

GRs are expressed in most brain cells and the glucocorticoids access different brain areas equipotently. As a consequence the specific cellular targets of the effects of GR activation on normal and pathological behaviors remain largely unknown. Identifying the specific cellular targets of GR effects on behavior is of the utmost importance. Thus, the molecular effects of GR largely vary as a function of the cellular type. Consequently, molecular mechanisms of glucocorticoid-mediated pathologies can only be understood once the specific cellular targets of these hormones have been identified.

In order to address this issue, using the tetracycline-regulated system (Tet-OFF system) [12]–[15], we developed an inducible transgenic approach with which a mutated form of the GR, called ΔGR can be expressed in specific brain areas. Compared to the wild-type GR, ΔGR lacks the Hormone Binding Domain (HBD) and the AF2 transcriptional activation domain [16], [17] and has a nuclear localization sequence (nls) instead that confers two essential properties: (i) ΔGR is mainly expressed in the nucleus and (ii) it is constitutively active and highly specific for the Glucocorticoid Responses Elements (GRE) [8]. As a consequence ΔGR overexpression reproduces the transcriptional effects of GR activation independently of glucocorticoid presence. Therefore ΔGR can be seen as a GR-molecular agonist with which GR-mediated transcriptional effects of stress in a specific cellular target can be reproduced *in vivo*. This approach bypasses several biases introduced by overexpressing the wild-type GR and submitting the animals to an actual stress. Thus in the latter case glucocorticoids levels need to be increased to activate the overexpressed wild-type GR. It will then be impossible to eliminate the influence of: 1. GR-independent effects of glucocorticoids; 2. Transcription-independent effects of GC-activated GR; 3 The effects mediated by the activation of the endogenous GR in other cellular types [18], [19].

In this report we used a transgenic approach that allows expression of the ΔGR prevalently in glutamatergic neurons of the dentate gyrus (DG) of the hippocampus. In these mutant animals ΔGR overexpression was stably induced at five months of age. These animal models then mimic the effects of certain forms of chronic stress specifically in this neuronal population in which the circadian secretion of glucocorticoids is lost and glucocorticoid levels are permanently high [20], [21].

In these animals we investigated anxiety-related behaviors, using the elevated plus maze and the emergence test. We also analyzed other GR-mediated behaviors that might indirectly modify performances in anxiety tests. We also studied the MAPK signaling pathway and the downstream MAPK-regulated protein Egr-1 since in the hippocampus they are regulated by the GR.

## Results

### Transgenic model for selective inducible overexpression of ΔGR *in vivo*

**Figure 1:**
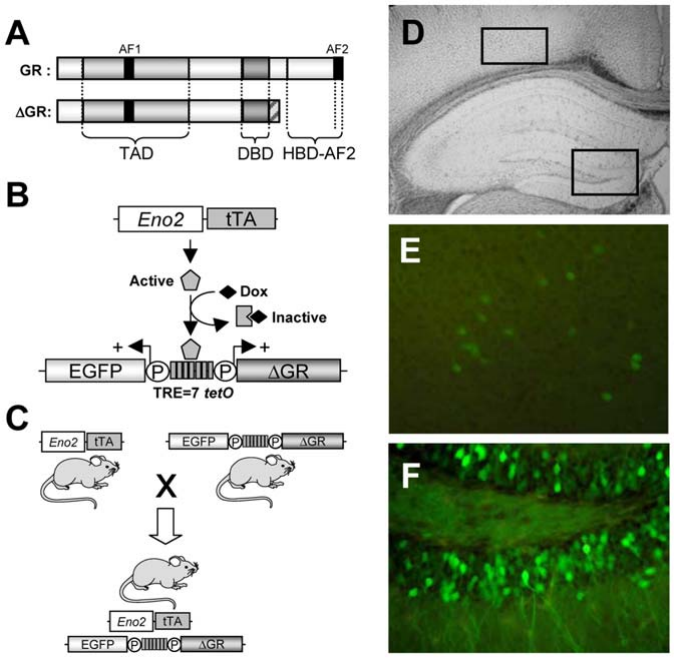
Expression pattern of EGFP and ΔGR proteins using the Tet-OFF system in genetically-modified mice. (**A**) ΔGR differs from the endogenous GR by an absence of the hormone binding and AF2 transcription domains which have been replaced by a C-terminal nuclear localization sequence to direct nuclear expression. (**B**) Schematic representation of the inducible Tet-OFF system. tTA expression is driven *in vivo* by the Enolase (*Eno2*) promoter. In absence of doxycycline (Dox), tTA protein binds to seven TetO sequences (TRE) to drive the co-transcription of EGFP and ΔGR transgenes. (**C**) Strategy to obtain Dox-dependent co-expression of EGFP and ΔGR proteins *in vivo* in double transgenic *Eno2*-ΔGR/EGFP mice. (**D**) EGFP expression was observed in coronal brain sections from *Eno2*-ΔGR/EGFP mice maintained in doxycycline-free conditions. (**E**) Few cells with weak EGFP expression were observed in the cortex. (**F**) Strong EGFP expression in the dentate gyrus of the hippocampal formation.

The selective inducible overexpression of ΔGR (Figure 1A, [8]) was obtained using the tetracycline-controlled transactivator (tTA)-regulated system (Tet-OFF system). We used a bidirectional construct allowing the co-expression of the Enhanced Green Fluorescent Protein (EGFP) and ΔGR under the control of Tetracycline Response Elements (TRE), which can be activated by the tTA protein in the absence of tetracycline's analogue doxycycline (Dox) [8], [12] (Figure 1B). The transgenic mice integrating this bidirectional construct (Tet-ΔGR/EGFP) were then crossed with regulatory mice in which the tTA transgene was controlled by the *Eno2* (Neuron Specific Enolase: NSE) promoter [12], [13] (Figure 1C).

### Anatomical and cellular localization of EGFP and ΔGR transgene expression *in vivo*

We first studied transgene expression by analyzing the pattern of EGFP expression in *Eno2*-ΔGR/EGFP mice maintained in doxycyline-free condition from birth to adulthood. We found only a few scattered EGFP positive cells in layer 5/6 in the cortex (Figure 1D, E) and a strong expression in the dentate gyrus of the hippocampal formation (Figure 1D, F).

**Figure 2:**
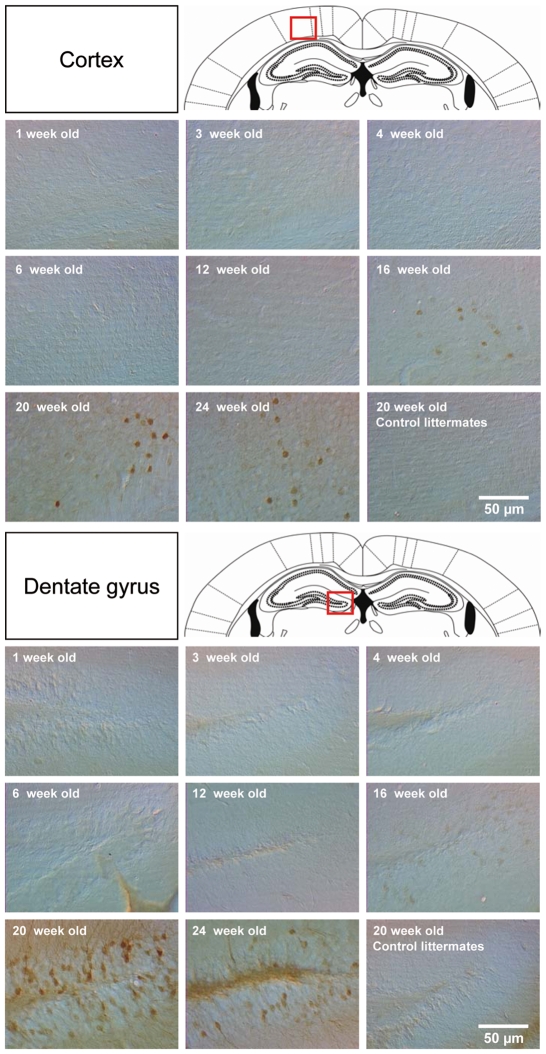
Spatial and temporal pattern of expression of EGFP transgene from 1 week to 24 week-old bigenic *Eno2*-ΔGR/EGFP mice. Schematic diagrams taken from the Paxinos and Watson atlas (1986), show the representative pictures for the cortical and dentate gyrus regions (red frame). 20 week-old control littermates were used as an immunohistochemical negative control.

A time course of the expression of the transgenes in *Eno2*-ΔGR/EGFP mice raised in doxycycline-free conditions revealed that the transgenes were only expressed in adulthood, starting at four months of age with maximum expression at around five months of age (Figure 2). Thus, for the following sets of experiments, *Eno2*-ΔGR/EGFP mice were maintained in doxycycline-free conditions and studied between five and six months of age, which corresponds to the optimal period of transgene activation. For each experiment transgene activation was verified before the start of the experiments and after the end of the experiments in all animals.

**Figure 3:**
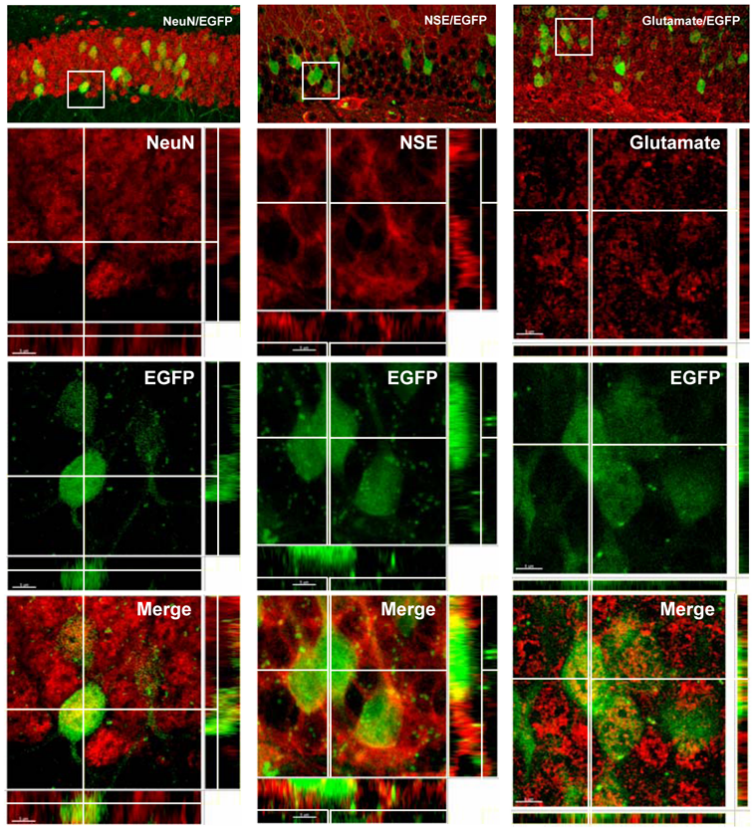
Cellular characterization of cells expressing EGFP and ΔGR proteins in the dentate gyrus of *Eno2*-ΔGR/EGFP bigenic mice. Confocal illustrations of neurons from the dentate gyrus co-expressing EGFP protein and specific neuronal markers visualized with Cy^3^-conjugated antibodies. Distribution of EGFP and endogenous neuronal markers (NeuN, NSE, Glutamate) and merges of the two signals are shown.

The phenotype of cells co-expressing EGFP and ΔGR proteins was analyzed using immunocytochemistry (Figure 3). In the DG, EGFP was present in mature neurons expressing the NeuN marker and in cells expressing the Neuron-Specific Enolase (NSE). This result indicated that the tTA protein under the control of the *Eno2* promoter used to induce EGFP and ΔGR transgenes is highly specific to this neuronal subtype. The transgene seemed to be selectively expressed by glutamatergic neurons since the EGFP protein was also coexpressed with Glutamate (Figure 3). Similar results were obtained for the few neurons that expressed EGFP in the cortex (Supplementary Figure S1).

**Figure 4:**
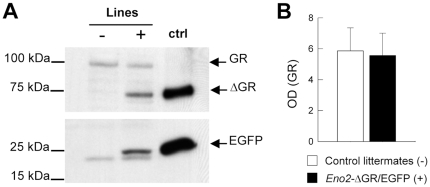
Expression of EGFP and ΔGR and GR proteins in the hippocampus. (**A**) Western blotting showing EGFP and ΔGR proteins in extracts from hippocampi dissected from control littermates (−) and *Eno2*-ΔGR/EGFP mice (+). Positive controls (ctrl) were obtained from protein cell extracts from CHO-K1 Tet-ON cells transfected with EGFP-TetO-ΔGR expression vector [8]. (**B**) Nuclear wild-type GR protein from hippocampi of *Eno2*-ΔGR/EGFP bigenic mice (n = 11) and control littermates (n = 7) were analyzed by western blot and quantified by densitometry (optical density, OD, means +/− sem).

ΔGR expression was studied using western blotting since this protein cannot be distinguished from the wild-type GR using immunohistochemistry. Thus, the ΔGR has the same structure as the wild-type GR except for the lack of hormone binding and the AF2 transcriptional activation domains. In contrast, the smaller ΔGR is easily detectable by western blotting. We found an expression of EGFP and ΔGR proteins in the hippocampus of bigenic mice (Figure 4A) with no modification in the quantity and the distribution of the endogenous GR (Figure 4B, t_16_ = 0.139 p>0.889). These findings are consistent with the immunohistochemistry results. EGFP and ΔGR were not detectable by western blotting in the cortex of bigenic mice. This is not surprising given the low number of positive cells and the low level of expression in this structure found using immunohistochemistry.

This very restricted spatial and temporal pattern of expression of this line of Tet-ΔGR/EGFP transgenic mice is probably due to the site of integration of the transgene within the genome. In addition it could also be due to the insertion of a low number of copies of the bidirectional construct into the genome [22], [23]. In order to explore the latter possibility, we determined the number of copies using real time quantitative PCR (qPCR) [24], [25] and found that these transgenic mice inserted only 2 copies of the bidirectional Tet-ΔGR/EGFP construct (Supplementary Figure S2).

### Glucocorticoid secretion in Eno2-ΔGR/EGFP bigenic mice

**Figure 5:**
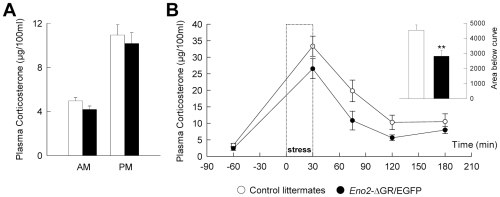
Basal and stress-induced corticosterone secretion in *Eno2*-ΔGR/EGFP bigenic mice. (**A**) Plasma corticosterone concentrations were determined in basal conditions from blood collected either one hour after light on (AM = 8 am) or one hour after light off (PM = 8 pm), these two time points correspond respectively to the lowest and highest levels of corticosterone during circadian secretion. Control (n = 15) and *Eno2*-ΔGR/EGFP bigenic mice (n = 10). (**B**) Kinetics of glucocorticoid secretion in response to stress (30 minutes of forced exposure to an open field). Mice were bled several times related to each time point and blood samples were collected from control (n = 13) and *Eno2*-ΔGR/EGFP bigenic mice (n = 10) 60 minutes before the beginning of the stress (t-60), at the end of the stress (t30), then 75, 120 and 180 minutes after the beginning of the stress. Insert represents the area under the curve; ** = *P*<0.01. Plotted values are means +/− sem.

We then analysed the potential modification of the HPA axis in *Eno2*-ΔGR/EGFP mutant mice. In order to analyze corticosterone secretion during the circadian cycle, plasma samples were collected 1 hour after lights on and 1 hour after lights off. These two time points correspond to the lowest and highest levels of corticosterone circadian secretion respectively [26]. Bigenic mice overexpressing ΔGR and their control littermates did not differ in terms of corticosterone secretion during the circadian cycle (Figure 5A, AM: t_23_ = 1.729 p>0.097; PM: t_23_ = 0.543 p>0.59) or the weight of the adrenal gland (data not shown). We also analyzed corticosterone secretion following acute stress (Figure 5B). In both control and mutant animals, 30 minutes of stress increased corticosterone levels. Over the following two hours, corticosterone secretion progressively returned to basal levels. Stress-induced corticosterone secretion was significantly lower in bigenic animals (Group effect: F_1,20_ = 5,00 p<0.037) as also shown by the analysis of the area under the curve (Figure 5B, inset: t_21_ = 3.068 p<0.006).

### Behavioral phenotypes of Eno2-ΔGR/EGFP bigenic mice

#### Anxiety-related behaviors

**Figure 6:**
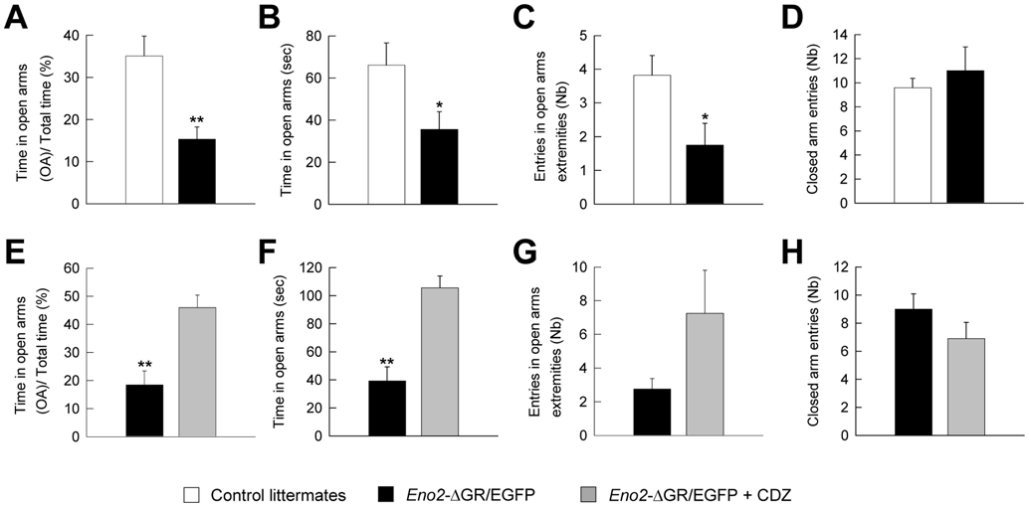
Anxiety-related behaviors in *Eno2*-ΔGR/EGFP bigenic mice. (**A–D**) Anxiety-related behavior was assessed with the elevated plus maze (EPM) test. Compared to control littermates (n = 11), *Eno2*-ΔGR/EGFP bigenic mice (n = 8) spent less time in the open arms section (**A, B**) and made fewer entries into the open-arm extremities (**C**) than into the closed arms (**D**). (**E–H**) In comparison to *Eno2*-ΔGR/EGFP bigenic mice treated with vehicle (Black bars, n = 4), intraperitoneal injection of chlordiazepoxide (CDZ; 7.5 mg/kg/ip) to *Eno2*-ΔGR/EGFP bigenic mice (Gray bars, n = 5) 15 minutes before the EPM test completely reversed this phenotype increasing the time spent in the open arms (**E, F**) and the entries in the arms extremities (**G**). Motor activity measured by entries into the closed arms did not differ between the two groups (**H**). Values shown are means +/− sem. * = *P*<0.05; ** = *P*<0.01.

The Elevated Plus Maze (EPM) is one of the most widely used tests for evaluating anxiety-related behavior. In this test, the animal is placed in the center of an elevated cross and can choose to walk in any of the four arms of the maze. Two of the opposite arms do not have walls (open arms) and are considered by mice as a threatening area. We used the time spent and number of entries into the open arms as a measure of anxiety [27] and into closed arms as a measure of locomotor activity [28]. *Eno2*-ΔGR/EGFP bigenic mice spent less time and visited the open arms and their extremities less often than control littermates (Figure 6A–C, A: t_17_ = 2.993 p<0.0087; B: t_17_ = 2.128 p<0.049; C: t_17_ = 2.273 p<0.037) suggesting an increase in anxiety-related behaviors. Changes in open arm exploration were not secondary to non-specific modifications of locomotor activity since closed arm entries were not modified between the mutant and control mice (Figure 6D, t_17_ = 0.784 p>0.444).

In order to further verify these findings we studied the effects of the reference anxiolytic, benzodiazepine chlordiazepoxide (CDZ) on *Eno2*-ΔGR/EGFP bigenic mice. The administration of chlordiazepoxide (CDZ, 7.5 mg/kg/ip) to *Eno2*-ΔGR/EGFP bigenic mice largely reversed the anxiety-related phenotype observed in *Eno2*-ΔGR/EGFP bigenic mice treated with vehicle (Figure 6E–G, E: t_7_ = 4.102 p<0.0045; F: t_7_ = 4.840 p<0.0019; G: t_7_ = 1.724 p>0.127). These results were not due to the non-specific effects of the mutation or of the pharmacological treatments since the number of entries in the closed arms did not differ between groups (Figure 6H, t_7_ = 1.055 p>0.325).

**Figure 7:**
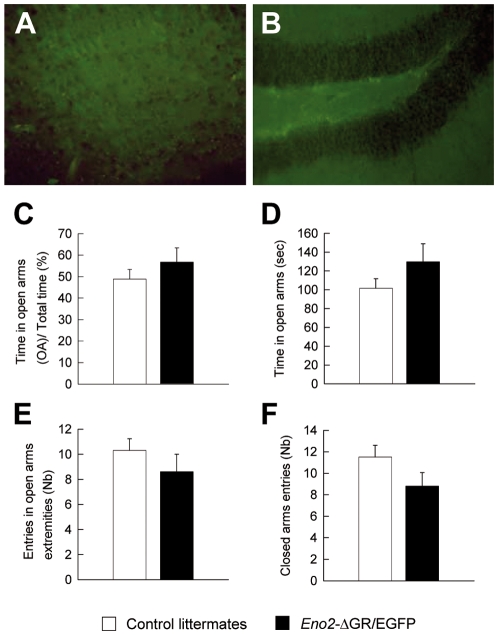
Anxiety-related behavior assessed in the EPM test on three month-old *Eno2*-ΔGR/EGFP bigenic mice. Three month-old *Eno2*-ΔGR/EGFP bigenic mice did not express EGFP and ΔGR transgenes in the cortex (**A**) and in the DG (**B**) and did not display anxiety-related behavior (**C–F**). Compared to control littermates (n = 10), *Eno2*-ΔGR/EGFP bigenic mice (n = 10) spent the same amount of time in the open arms section (**C–D**) and made equal entries into the open arms extremities (**E**) than into the closed arms (**F**). Values shown are means +/− sem.

In order to analyze whether the observed phenotype was due to the expression of the ΔGR we studied anxiety-related behavior in the EPM in three month-old *Eno2*-ΔGR/EGFP bigenic mice, i.e. before these mice expressed the ΔGR transgene (Figure 7A–B). No behavioral differences in the EPM were found between *Eno2*-ΔGR/EGFP bigenic mice and control littermates (Figure 7C–F, C: t_18_ = −0.983 p>0.337, D: t_18_ = −1.288 p>0.212, E: t_18_ = 1.010 p>0.324, F: t_18_ = 1.606 p>0.124). These results suggest that the increase in anxiety-related behaviors observed in *Eno2*-ΔGR/EGFP bigenic mice at five months of age (Figure 6) is due to the expression of the ΔGR.

**Figure 8:**
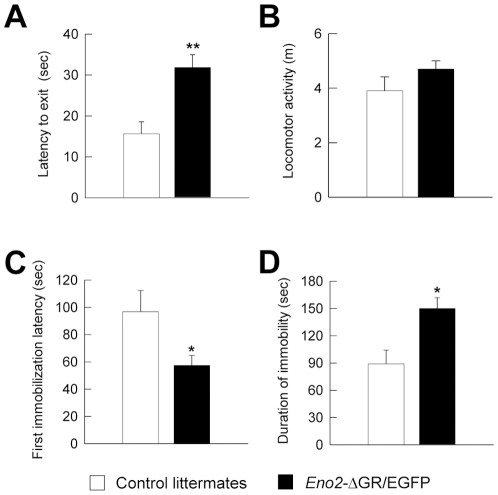
Anxiety- and depression-related behaviors in *Eno2*-ΔGR/EGFP bigenic mice. Anxiety-related behavior was measured in the emergence test. The latency to emerge from the dark cylinder was significantly longer in *Eno2*-ΔGR/EGFP (n = 10) than in control littermate (n = 13) (**A**). However, motor activity was comparable once outside the cylinder (**B**). In the forced swim test, which is commonly used to screen for antidepressant, the latency to the first immobilization was significantly lower (**C**) and the duration of immobility was increased (**D**) in *Eno2*-ΔGR/EGFP mice (n = 9) than in control littermates (n = 7). Values shown are means +/− sem. * = *P*<0.05; ** = *P*<0.01.

We extended these studies by analyzing the behavior of *Eno2*-ΔGR/EGFP bigenic mice in the emergence test (Figure 8A, B) which is also widely used to analyze anxiety-related behavior. In this test, the animal is placed in an opaque plastic cylinder located in a brightly lit open field. The cylinder and the open field are respectively a protective and a threatening environment for the mice. *Eno2*-ΔGR/EGFP bigenic mice had significantly longer latencies before emerging from the protective cylinder, indicating higher anxiety when faced with the threatening open field (Figure 8A, t_21_ = −3.706 p<0.0014). These results were not due to a non specific impairment of motor behavior since once out of the cylinder there was no difference in total motor activity between the two strains (Figure 8B, t_21_ = 1.497 p>0.148).

We then analyzed the behavior of *Eno2*-ΔGR/EGFP mice in the forced swim test (Figure 8C, D) which is the most widely used behavioral measure to screen for antidepressant drugs [29]. However, in animal models anxiety and depression are not two dimensions that can be easily separated and most tests actually screen different forms of behavioral responses to unavoidable aversive situations. In the forced swim test, mice are forced to swim in a small transparent cylinder. After unsuccessful attempts to escape, mice stop swimming and float. We measured both the latency and the duration of immobility as a measure of despair (learned helplessness) [29]–[31]. *Eno2*-ΔGR/EGFP bigenic mice showed a lower latency to the first immobilization (Figure 8C, t_14_ = 2.650 p<0.021) and increased duration of immobility compared to control littermates (Figure 8D, t_14_ = −2.797 p<0.015).

**Figure 9:**
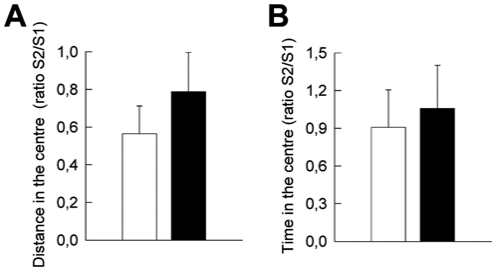
Novelty-induced explorations in *Eno2*-ΔGR/EGFP bigenic mice. Exploration of a novel stimulus was performed in a familiar environment using the novel object test. The S2/S1 ratio is respectively a measure of (**A**) the distance covered or (**B**) the time spent in the centre of the apparatus in presence of the object in session 2 (S2) over the distance or the time spent in the centre of the apparatus without the novel object during session 1 (S1). These ratios indicate that the novel object increased exploration similarly in both genotypes. Control littermates mice (n = 9), *Eno2*-ΔGR/EGFP bigenic mice (n = 11). Values shown are means +/− sem.

The time spent in novel open areas, usually used to measure anxiety, results from the computation of two opposite motivational forces. The fear of potential threats driving avoidance, and novelty-seeking driving exploration. Consequently, a decrease in the time spent in the open arms of the EPM or an increase in the latency in exiting the protective cylinder in the emergence test could result from either an increase in the fear of potential threats or a decrease in novelty exploration. In order to address this issue, we evaluated *Eno2*-ΔGR/EGFP bigenic mice for the exploration of a novel object in a non-threatening environment [32]. For this test animals are first habituated to the test apparatus in the absence of the object in order to diminish the fear component of exposure to an unknown environment (session 1: S1). Then, in a subsequent session a novel object is added (session 2: S2). Exploration of the novel object is measured by comparing the distance (Figure 9A) and the time spent (Figure 9B) into a zone of the open field in the presence or in the absence of the novel object. In these conditions, it was found that exploration of a novel object (Figure 9, A: t_18_ = −0.835 p>0.413; B, t_18_ = −0.324 p>0.748) did not differ between bigenic and control littermates.

Taken together these results indicate that *Eno2*-ΔGR/EGFP bigenic mice have an increase in stress-related behavior and in particular in behaviors suggesting higher anxiety and despair induced by aversive situations.

#### Circadian and novelty-induced locomotor activation

**Figure 10:**
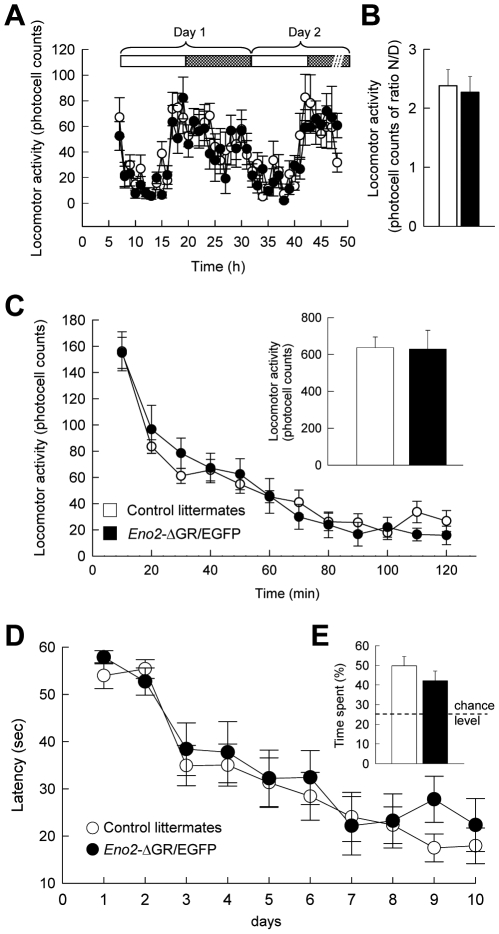
Circadian and novelty-induced locomotion and spatial memory in *Eno2*-ΔGR/EGFP bigenic mice. (**A**) Recording of locomotor activity, measured by photocell beam breaks over two days did not reveal any differences between mutants (n = 8) and control mice (n = 11). White and dashed boxes represent the light and dark cycle respectively. (**B**) The amplitude of the circadian rhythm measured by the ratio between locomotor activity during the light and dark periods was not different between *Eno2*-ΔGR/EGFP bigenic mice and control littermates. (**C**) Novelty-induced locomotor activity was measured by photocell beam breaks during two hours of forced exposure to a novel activity box. Inset: total activity during the two hour period. No differences in locomotor activity were observed between control littermates (n = 13) and *Eno2*-ΔGR/EGFP bigenic mice (n = 9). (**D**) Spatial learning in *Eno2*-ΔGR/EGFP bigenic mice was tested in the Morris water maze test. Mice have to find a hidden platform using extra maze cues. Latency to reach the platform from variable start positions decreased over time similarly in mutants (n = 10) and control mice (n = 14). (**E**) During the probe test, the platform is removed from the pool and the time spent in the target quadrant, the one previously containing the platform, is measured. No differences were observed between *Eno2*-ΔGR/EGFP bigenic mice (n = 10) and control littermates (n = 14). Values shown are means +/− sem.

Glucocorticoid hormones have been involved in the regulation of locomotor activation. In order to further verify that ΔGR overexpression in the DG did not modify locomotor activity in *Eno2*-ΔGR/EGFP we studied the increase in motor activity observed during the active phase of the circadian cycle and induced by a mild stress, such as the forced exposure to a novel environment. Both these behaviors are believed to involve the activation of the GR by glucocorticoids [6]. We found that ΔGR overexpression did not modify locomotor activity during the circadian cycle (Figure 10A, Group x Time interaction: F_41,902_ = 0.916 p>0.621) and had no effect on the Night/Day ratio, an index of the rhythmic activity (Figure 10B, t_17_ = −0.078 p>0.937). In addition, there was no difference in novelty-induced locomotion between bigenic and control mice (Figure 10C, Group x Time interaction: F_11,209_ = 1.073 p>0.383; inset: t_20_ = 0.085 p>0.931).

#### Spatial memory

Activation of the GR by glucocorticoid hormones has also been been implicated in the regulation of learning and memory and in particular spatial memory [33] a cognitive function that strongly involves hippocampal formation [34]. Hippocampus-dependent spatial navigation was studied here using the water maze task. In this behavioral procedure the animal has to learn the location of a hidden platform using distal cues while the starting position is changed at each trial. This procedure requires the hippocampus since the animal has to learn the positional relations among multiple independent environmental cues (“spatial relational memory”) in order to find the hidden platform. *Eno2*-ΔGR/EGFP bigenic mice and their control littermates showed similar learning of the location of the platform during training (Figure 10D, Group x Time interaction: F_9,198_ = 0.523 p>0.855). These results were confirmed by a probe test, in which the hidden platform is removed. This procedure measures over 60 seconds the time spent by the animal in the quadrant where the platform was located during training (target quadrant). Both strains showed a similar memory of the platform location during a probe test, spending more than 40% of their time in the target quadrant (Figure 10E, t_22_ = 1.077 p>0.292). Altogether, these results indicate that the overexpression of the ΔGR did not modify spatial memory.

### MAPK signaling and Egr-1 up-regulations in Eno2-ΔGR/EGFP bigenic mice

**Figure 11:**
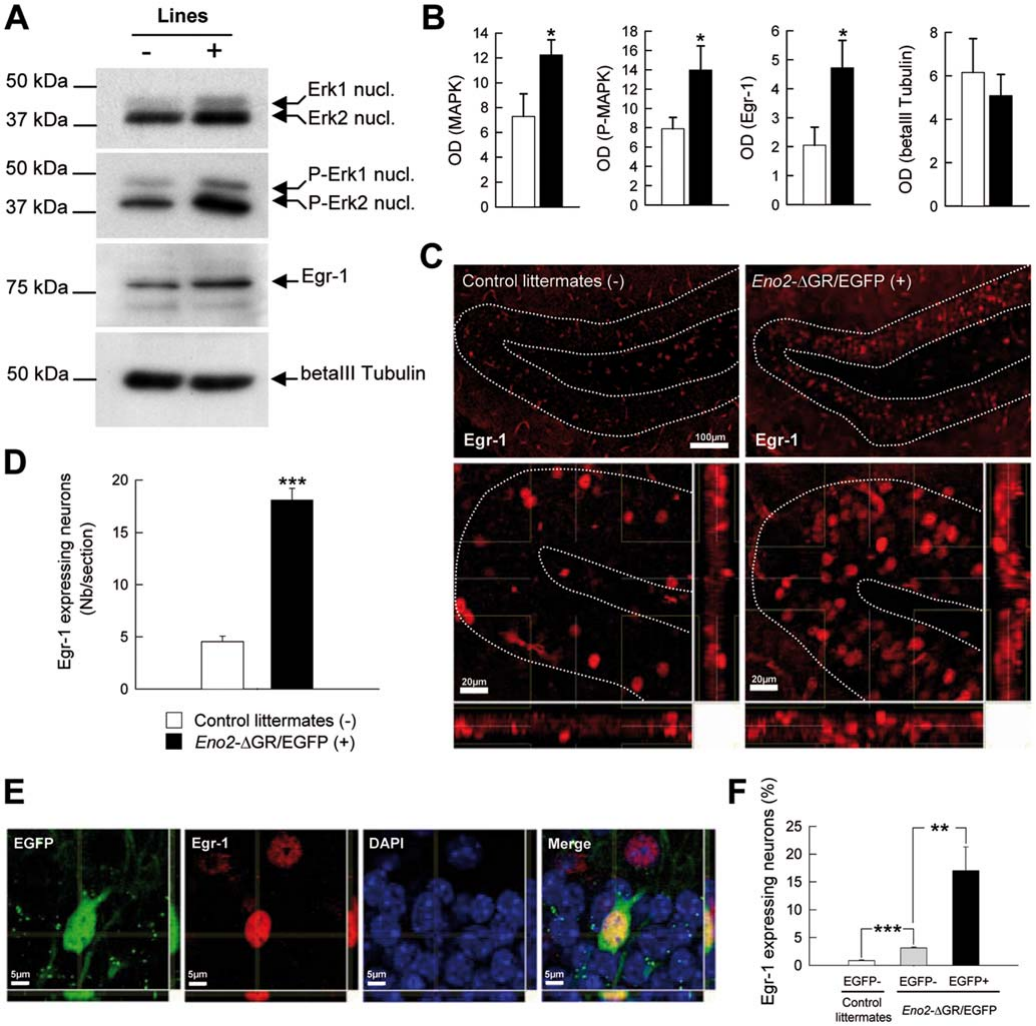
Stimulation of the MAPK pathway and Egr-1 by ΔGR in *Eno2*-ΔGR/EGFP bigenic mice. (**A**) Nuclear non-phosphorylated, phosphorylated Erk1/2 and Egr-1 proteins from hippocampi of *Eno2*-ΔGR/EGFP bigenic mice (+) (n = 9) and control littermates (−) (n = 9) were analyzed by western blot. BetaIII-tubulin was used as a loading control. (**B**) The corresponding X-Ray films were quantified by densitometry (optical density, OD, means +/− sem) and showed a higher expression of the proteins studied in *Eno2*-ΔGR/EGFP bigenic mice. (**C**) Confocal illustration of neurons expressing Egr-1 within the dentate gyrus of *Eno2*-ΔGR/EGFP bigenic mice and control littermates. (**D**) Number of Egr-1 expressing neurons in the dentate gyrus of *Eno2*-ΔGR/EGFP bigenic mice (black, n = 4) and control littermates (white, n = 4). (**E**) Confocal illustration of neurons co-expressing EGFP (green) and Egr-1 (red) in the dentate gyrus of *Eno2*-ΔGR/EGFP bigenic mice; the cells were counterstained with DAPI (blue). (**F**) Percentage of Egr-1 expressing neurons within the different subpopulation of cells in the dentate gyrus of *Eno2*-ΔGR/EGFP bigenic mice and control littermates. Values shown are means +/− sem. * = *P*<0.05, ** = *P*<0.01, *** = *P*<0.001.

We finally investigated whether ΔGR overexpression in the hippocampus leads to alteration in expression and activity of target genes that are known to be modulated by GR activation in this structure. For this purpose we studied a key member (Erk1/2) of the MAPK signaling pathway and the downstream-regulated Egr-1 protein. Both Erk1/2 phosphorylation and Egr-1 transcription are activated by the GR and involved in the behavioral response to threatening stimuli [8]. Both nuclear phosphorylated and unphosphorylated Erk1/2 as well as Egr-1 proteins were up-regulated in the hippocampus of *Eno2*-ΔGR/EGFP bigenic mice (Figure 11A, B, MAPK: t_16_ = −2.747 p<0.0144; P-MAPK: t_16_ = −2.194 p<0.0434; Egr-1: t_16_ = −2.882 p<0.02; betaIII tubulin: t_16_ = 0.714 p>0.485). Further immunohistochemical analysis showed that Egr-1 expression was increased in the DG of *Eno2*-ΔGR/EGFP bigenic mice (Figure 11C, D; t_6_ = −6.293 p<0.0008) and prevalently within the neuronal subpopulation expressing the ΔGR (Figure 11E, F; *Eno2*-ΔGR/EGFP mice versus control for Egr-1 in EGFP negative cells: t_6_ = −6.697 p<0.00054; Egr-1 in EGFP positive versus EGFP negative cells in *Eno2*-ΔGR/EGFP mice: t_6_ = −4.672 p<0.00343).

## Discussion

In this report, using the tetracycline-regulated system (Tet-OFF), we created a conditional transgenic mouse line that expresses a nuclear constitutively active form of the GR (ΔGR) prevalently in glutamatergic neurons of the dentate gyrus (DG) of the hippocampus. ΔGR lacks the hormone-binding and AF2 transcriptional activation domains and is able to activate GR-mediated transcription in the absence of glucocorticoids. Thus, the transcriptional effects of selectively activated GR in the target population of neurons can be mimicked with this protein. DG-*Eno2*-ΔGR/EGFP bigenic mice displayed enhanced stress-related behaviors and in particular higher anxiety- and depression-related behaviors in response to unavoidable aversive situations. Conversely, other behaviors, such as novelty exploration, locomotor activity and spatial learning, which could influence the performance in anxiety tests, were not modified. The behavioral phenotype in DG-*Eno2*-ΔGR/EGFP was associated with an up-regulation of the MAPK cascade and the downstream-regulated Egr-1 protein.

### A novel inducible transgenic strategy to study the role of the GR in a specific brain region

We generated several lines of ΔGR/EGFP mice. We observed that depending on the line, the expression pattern of EGFP in doxycycline-free conditions could vary and be different from the one described by Chen and coworkers [13] for the Enolase transgene. For example the line used in this paper (Line 27; L27) exhibited strong expression only at the adult stage within the dentate gyrus of the hippocampal formation, and very weak expression in a few cells in the cortex. On the other hand, a second mouse line we generated (Line 23; L23) did not express the ΔGR in the DG, whilst it showed strong expression in the cortex, the dorsal and ventral striatum, and the CA1 of the hippocampus (Supplementary Figure S3).

This variability in transgene expression is caused mainly by the stochastic event of transgene integration within the host genome and the nature of the transgenic constructs (i.e. minimal promoter). It is well accepted that host sequences surrounding the site of transgene integration but also transgene copy numbers, methylation at the transgene locus and heterochromatin-induced position effect variegation (PEV) can modify the expected expression pattern, potentially causing it to be ectopic, weak, delayed or even undetectable. This is currently interpreted as the result of chromosomal position effects [22], [23], [35]–[37]. These caveats however also provide the possibility of generating mice strains with partially overlapping and distinct patterns which can be used to study the role of a target protein, in our case the GR, in selective brain structures.

### Behavioral effects of the transcriptional activation of the GR in the Dentate Gyrus

The hippocampal formation is known to be involved in most of the glucocorticoid-mediated behaviors studied in this report. Our results indicate that the selective activation of GR in glutamatergic neurons of the DG is a sufficient condition to modify anxiety-related behaviors, as measured by EPM, emergence and forced swim tests. The relationship of the phenotypes observed in animals overexpressing the ΔGR in the DG with anxiety is strengthened by the observation that treatment with the prototypical anxiolytic CDZ completely abolished the enhanced response of these animals in the EPM.

It is noteworthy that the forced swim test has also been linked to depression and is largely used to screen for antidepressants. Several data indicate that anxiety disorders share common symptoms with depression and anxiety and depression frequently coexist [38]. Furthermore, most antidepressants also have anxiolytic effects [39]. This is probably why overexpression of the ΔGR in a specific cellular target modified both prototypical anxiety-related behaviors and the forced swim test.

The very restricted expression of ΔGR in the mouse line used in these experiments suggests that the phenotype observed is likely due to the over-activation of the ΔGR in the DG. This idea is also supported by behavioral results we obtained in another mouse line (Line 23) which expressed the ΔGR in several brain structures but not in the DG (Supplementary Figure S3). Thus, in Line 23 the elevated plus maze, the emergence test and the forced swim test were not modified (Supplementary Figure S4).

A prominent role of the DG in some of the hippocampus-mediated behaviors is consistent with the anatomical position of this structure in the hippocampal circuitry since the DG is the entry point for afferences to the hippocampus which receives its major inputs from the cortex [40].

The finding of increased anxiety and despair in ΔGR mice is also consistent with previous publications. It has been shown that mice in which the *gr* gene has been knocked out in the whole brain demonstrate a decrease in anxiety-related behaviors as measured by the zero maze, a variant of the EPM [41], [42]. Conversely, an overexpression of wild-type GR in the entire forebrain (GRov) has been observed to induce an increase in anxiety-related behaviors in the EPM and a shorter latency to immobilization in the forced swim test [43].

Our findings extend these previous observations by showing that the selective activation of GRs selectively in the glutamatergic neurons in the DG is a sufficient condition to induce these behavioral phenotypes. They also indicate that the hormone binding and the AF2 transcriptional activation domains of the GR molecule, lacking in the ΔGR are not the structural domains involved in the establishment of these stress-related behaviors in the DG.

It has previously been suggested, using hippocampal lesions, that the hippocampus is also involved in anxiety-related behavior [44], [45]. Our data highlight an important role for the DG in anxiety-related processes. Our findings are in line with three recent reports. First, it has been shown that the suppression of neural activity in the DG reverses the anxiety-related phenotype of *^Htr1a^*KO mice [46]. Secondly, selective inhibition of neurogenesis in the dentate gyrus of the hippocampus has been found to induce a strong increase in anxiety-like behaviors [47]. Thirdly, mice in which the GR has been disrupted using a lentivirus-based strategy in the central nucleus of the amygdala (CeA), did not display a decrease in innate fear but showed deficits in fear conditioning [48]–[50]. The latter observation suggests that GR in the CeA, a structure strongly implicated in anxiety-related process [48]–[50], may be implicated in learned fear, whilst GR in the DG could mediate innate fear responses like those measured in the EPM and emergence tests [51], [52].

### Specificity of the behavioral effects of the GR in the Dentate Gyrus

Overexpression of ΔGR in the DG seems to modify anxiety-related behavior in a specific way. Thus other behaviors such as novelty seeking, motor activity and spatial memory that could indirectly modify performances in anxiety-related tests were unchanged in our experimental conditions.

The lack of these effects is not all that surprising. Although the hippocampus seems to play a role in modulating novelty seeking, this behavior is thought to be controlled mainly by the dopamine circuit in the basal ganglia, a brain region that did not express ΔGR in our mouse line. In parallel, the regulation of circadian activities by the GR involves several brain regions at the same time [53] and consequently could be unaffected by the selective modification of GR activity in the DG. The results concerning spatial memory might seem more surprising since both the GR and the hippocampus have been implicated in the regulation of this behavior [33], [34], [54], [55]. One explanation for these discrepancies is that a larger impact on the hippocampus is probably necessary to modify spatial memory and that restricted modification of the GR in DG glutamatergic neurons is not sufficient.

Finally, the modification in anxiety-related behavior observed in our study seems to be independent of an increase in corticosterone secretion that could modify the activity of other brain structures. Thus, transgenic mice did not exhibit any alteration in basal circulating plasma corticosterone levels and showed reduced stress-induced corticosterone secretion. Although these results may seem surprising, they are in agreement with what has been found using mice overexpressing full-length GR which display unmodified corticosterone secretion during the circadian cycle [43] and decreased corticosterone secretion after stress [56]. This dumped stress-induced corticosterone secretion is probably due to an increase in GR-mediated negative feedback that inhibits stress-induced corticosterone [57].

### Downstream signaling pathway mediated by GR activation in the hippocampus

Increased ΔGR expression in the DG was also associated with an increase in the expression and enzymatic activity of the MAPK signaling pathway which resulted in increased expression of the zinc finger transcription factor Egr-1.

These results are important for several reasons. First, they show at a molecular level that overexpression of the ΔGR in the DG is functionally active *in vivo* extending previous results obtained *in vitro* with the ΔGR [8]. Secondly, they shed some light on the potential mechanisms through which ΔGR overexpression could modify reactivity to threatening stimuli. Indeed, inhibition of the MAPK pathway has been shown to decrease glutamate release [58], [59]. Glutamate that is increased by stress [58], [60]–[65] through glucocorticoids in the hippocampus [63], [64], [66], [67], [67]–[69] has recently been shown to play an important role in stress responses and anxiety disorders [70]. Therefore, it seems reasonable to hypothesize that the increase in anxiety observed in ΔGR animals could be mediated by a MAPK-dependent increase in the release of glutamate.

### Conclusions

In conclusion our data provide evidence that the anxiety-related effects of glucocorticoid involve the activation of the GR in glutamatergic neurons of the DG of the hippocampus. Our results also restrict these behavioral modifications to transcriptional effects of the GR that do not need the hormone binding and the AF2 domains and point to an involvement of the MAPK signaling pathway and the downstream MAPK-regulated protein Egr-1. The identification of a neural target for anxiety-related effects of GR activation may open the way to underpin the precise molecular basis of certain stress-related disorders.

## Materials and Methods

### Animals

Tet-ΔGR/EGFP founder mice were amplified under C57BL/6J (Charles River, Lyon, France) genetic background. Mice expressing the transgene for the tetracycline transactivator (tTA) under the control of the Enolase (*Eno2*) promoter, kindly provided by Dr. E.J. Nestler (University of Texas Southwestern Medical Center) [13], were backcrossed for seven generations to maintain their homozygous state. Breeding homozygous *Eno2*-tTA and heterozygous Tet-ΔGR/EGFP mice yields 50% bigenic mice and 50% *Eno2*-tTA mice used as control littermates. A 12 hr light/dark cycle (lights on from 7am to 7pm) was used in the animal house. Food (SAFE: Scientific Animal Food and Engineering #A04, France) and water were available *ad libitum*. Animals were maintained in a temperature (22±1°C) and humidity (55±5%) controlled environment. All experiments were conducted in strict compliance with the European Communities Council Directive of 24 November 1986 (86/609/EEC), and approved by the Aquitaine-Poitou Charentes ethical committee.

### Plasmid construction and *in vivo* gene targeting

#### Transgenic construct

The pBI-EGFP-TetO-ΔGR vector used to generate transgenic animals transcribes two genes (*egfp* and Δ*gr*) from one bidirectional Tet-responsive promoter. It was obtained by sequentially cloning the *egfp* and the *Δgr* genes under the control of the Tet Response Elements (TRE) (Revest et al. [8] for a detailed description). The pBI-ΔGR-TetO-EGFP construct was then excised from the plasmid backbone by PshAI/HaeII digestion. Microinjection into fertilized (C57BL/6JxCBA) F_2_ oocytes and other surgical procedures were performed within the transgenic core facility at Bordeaux 2 University.

#### Genotyping

Genomic DNA was isolated from tail clips and blood and genotype determined using different sets of primers to discriminate between monogenic heterozygous *Eno2*-tTA and bigenic *Eno2*-ΔGR/EGFP mice. PCR protocols using Taq Polymerase (Biolabs, UK) to analyze tTA and ΔGR transgenes respectively were 95°C 1 min, then 35 cycles of 95°C 45 sec, 56°C 45 sec, 72°C 2 min, then 72°C 10 min; and 95°C 1 min, then 30 cycles of 95°C 45 sec, 65°C 45 sec, 72°C 3 min 30 sec, then 72°C 10 min.

Primer tTA forward: 5′-CGCTGTGGGGCATTTTACTTTAG-3′;

primer tTA reverse: 5′-CATGTCCAGATCGAAATCGTC-3′.

Primer ΔGR forward: 5′-tacccgggtcgagtaggcgtgtac-3′;

primer ΔGR reverse: 5′-GGCTTGATAAGATTGTATCTCCAG-3′.

The transgene copy number was evaluated using real time quantitative PCR (qPCR) by determining the threshold cycle (Ct) of the transgene and a standard curve generated from a serial dilution of known quantities of the pBI-ΔGR-TetO-EGFP cDNA plasmid [25]. Briefly, genomic DNA isolated from the tail by proteinase K digestion was used, followed by phenol-chloroform extraction to remove real-time PCR inhibitors. qPCR amplification used sets of specific primers to amplify both EGFP and actine genes.

Primer EGFP forward: 5′-GGGCACAAGCTGGAGTACAACTA-3′;

primer EGFP reverse: 5′-CCTTGATGCCGTTCTTCGC-3′.

Primer Actine forward: 5′-TGACCGAGCGTGGCTACA-3′;

primer Actine reverse: 5′-CATAGCACAGCTTCTCTTTGATGTC-3′.

All samples were run in triplicate using the Dynamo HS SYBR Green qPCR kit (FINNZYME, Espoo, Finland) according to the manufacturer's instructions [24]. PCR was run on a Opticon2 cycler (MJ Research/Biorad, Hercules, CA, USA) using the following amplification parameters 95°C 15 min, and 40 cycles at 95°C 20 sec and 61°C 35 sec. Fluorescence at each cycle was normalized to the reference dye and the parameter Ct (threshold cycle) was defined as the fractional cycle number above the background noise at which the fluorescence passes a fixed threshold. The copy number of genomic transgenes was calculated based on the following formula:

(http://www.appliedbiosystems.com/support/tutorials/pdf/quant_pcr.pdf) also including the mass of the haploid mouse genome (C-value) which is around 3.28 pg (http://www.genomesize.com).

### Blood collection for corticosterone assay

For all the experiments described below blood was collected through a small incision at the base of the tail vein made with a razor blade which allowed the collection of 30 µl of blood. Blood obtained via tail sampling was collected individually in capillaries coated with heparine-litium (Sarstedt, France) and centrifuged at 13,000 rpm (4°C, 10 min). Supernatant containing the blood plasma was stored at −20°C, and then processed for corticosterone assay.

**Circadian cycle experiment:** Blood samples from *Eno2*-ΔGR/EGFP and their control littermates were collected one hour after light on and one hour after light off. During the dark period, blood sampling took place under red light conditions.

**Stress experiment:** A first blood sample was taken 60 minutes (t_−60_ = basal condition) before the beginning of the stress (t_0_). Mutant and control male mice were then subjected to 30 minutes stress in a brightly lit (500 lux) square open field (50×50 cm×40 cm high). Twelve open fields were located in an isolated room and 12 mice were tested in parallel. The experimenter was not present in the room during the 30 minute period and was unaware of the experimental group. Immediately after the end of the 30 minutes stress procedure a blood sample was collected by a small incision of the tail (t_30_). Animals were then placed back into their home cages and blood samples were taken 75 (t_75_), 120 (t_120_) and 180 (t_180_) minutes after stress onset. Blood samples from the same animal were collected from 5 distal to proximal incisions of the tail vein corresponding to the 5 times studied.

### Corticosterone assay

Plasma corticosterone levels were measured by radioimmunoassay (RIA) as described elsewhere [19] using a highly specific corticosterone antiserum (MP Biomedicals, France). The minimum level of detection was 0.1 µg/100 ml, and the intra- and interassay coefficients of variation were approximately 4.5 and 10%, respectively.

### Immunohistochemistry

Mice were perfused transcardially with a phosphate-buffered solution of 4% paraformaldehyde and 0.25% glutaraldehyde in 0.1 M phosphate buffer at pH 7.4. After perfusion, 40 µm brain sections were cut on a vibratome and processed with a standard immunohistochemical procedure using specific primary antibodies. Free-floating sections were quenched with 0,5% NaBH_4_, 0,2% Na_2_S_2_O_5_ in 0.1 M phosphate-buffered saline at pH 7.4 for 20 minutes to remove the unbound excess of aldehydes, then washed 3 times with PBS containing 0,2% Na_2_S_2_O_5_ and then were processed according to a standard immunohistochemical procedure. Briefly, sections were subjected to 72 h of incubation at 4°C respectively: for NeuN-ImmunoReactivity (IR) using an anti-NeuN mouse monoclonal antibody (1/1000, Chemicon, USA); for NSE-IR using an anti-NSE (NSE: Neuron Specific Enolase) rabbit polyclonal antibody (1/250, Chemicon, USA); for Glutamate-IR using an anti-Glutamate mouse monoclonal antibody (1/1000, Gemacbio, France), for Egr-1 using a polyclonal rabbit anti-Egr-1 (1/500 Santa Cruz, USA). Sections were then incubated with Cy^3^ conjugated-secondary antibodies for 2 h at room temperature, then washed and mounted on Superfrost Plus slides (MenzelGmbH&Co KG, Braunschweig, Germany) with mowiol or ProLong Gold Antifade Reagent containing DAPI (Molecular Probes-Invitrogen, UK). Confocal microscopic imaging was performed using a Leica microscope (DMR TCS SP2 AOBS). For EGFP immunostaining, a standard immunohistochemical procedure was used [71]. Sections were incubated with a rabbit polyclonal anti-EGFP antibody (#8367-1; 1/500, Clontech). Immunoreactivities were visualized by the biotin-streptavidin technique (ABC kit, Dako) using 3,3′-diaminobenzidine as a chromogen. Microscopic imaging was performed using a Leica microscope DMRXA2 equipped with a Nomarski filter.

### Immunoblotting analysis

Protein extracts containing protease and phosphatase inhibitors from mice hippocampi were prepared using a procedure previously described and validated [19]. Proteins suspended in Laemmli buffer were separated by SDS-PAGE (10% gels), transferred onto PVDF membranes (Millipore, US) and revealed with specific antibodies. The following rabbit polyclonal antibodies were used: anti-GR (#sc-1004-X; 1/10000, Santa Cruz), anti-EGFP (#8367-1; 1/1000, Clontech), anti-Egr-1 (#sc-189; 1/500, Santa Cruz), anti-MAP kinase (#06-182; 1/200000, Upstate), anti-P-MAPK (#9101; 1/1000, Cell Signalling Technology). Eurogentec provided the Neuronal Class III β-Tubulin (TUJ1) monoclonal antibody (#MMS-435P, 1/20000). The X-Ray films were quantified by densitometry using a GS-800 scanner (in transmission mode) and the associated Quantity One software (Bio-Rad, CA, USA) following the manufacturer's instructions.

### Behavioral experiments

Behavioral experiments were conducted on mutant and control male mice, housed individually (dimension of the housing cage: length 29 cm; width 11 cm; height 13 cm) for the 15 days preceding the tests. All behavioral tests took place between 8am and 1pm. To eliminate odor cues, all testing equipment was thoroughly cleaned after each animal.

#### Elevated Plus Maze (EPM)

The apparatus consists in an elevated cross formed by two open arms (without walls, length = 45 cm, width = 5 cm) and two closed arms (length = 45 cm, width = 5 cm, height = 15 cm) made of Plexiglas radiating from a central platform to form a plus-sign. The apparatus was situated 51 cm above the floor. Brightness is adjusted to 100 lux for each area of the maze. Mice behavior was recorded by a video camera positioned above the maze and the number of entries into open and closed arms and the time spent on each arm were recorded (Vidéotrack, Viewpoint, Lyon France). The open arms are considered by mice as a threatening area. Animals were placed into the central area facing one open arm and allowed to explore the maze for 5 minutes. Percentage of time in open arms (OA) (time spent in open arms/(time spent in open + enclosed arms)x100), and time spent in OA and the number of closed and end-arm entries, were calculated [72]–[74]. To study the effects of anxiolytics, mutant mice were injected intraperitoneally with control solution or 7.5 mg/kg chlordiazepoxide (CDZ) [47]. Briefly, the benzodiazepine Chlordiazepoxide hydrochloride (CDZ, Sigma-RBI, USA) was dissolved with Cremophor EL (Sigma, USA) (1%) in sterile physiological saline (0.9% NaCl). Cremophor EL alone in saline solution was used as control solution. All injections were performed in a volume of 10 µl/g of body weight. Fifteen minutes after the injection, both groups were tested in the EPM.

#### Emergence test

This test was performed in a brightly lit (500 lux) open field (50×50 cm×40 cm high) containing an opaque cylinder (10-cm-deep and 6.5 cm in diameter) located lengthwise along one wall, with the open end 10 cm from the corner. During the test session which lasted 15 minutes the behavior was videotaped and then scored by a trained observer blind to genotype. At the beginning of the test session each mouse was placed in the cylinder and the latency to leave the cylinder (defined as placement of all four paws in the open field) and the locomotor activity in the open field were evaluated. The latency to emerge from the cylinder to go in the open space, which is a threatening area for the mice, is considered as an index of anxiety [32].

#### Forced swim test

In this test mice are forced to swim in a small transparent cylinder (19 cm in diameter and 25 cm high) filled with water (25°C, 20 cm deep) to avoid temperature-related stress responses. After unsuccessful attempts to escape, animals stop swimming and float. A mouse was judged immobile when it stopped all active behaviors (i.e. struggling, swimming, and jumping) and remained passively floating. Floating is considered as a measure of despair (learned helplessness) because the animals appear to stop trying to escape [29]. Behavior was recorded by a video camera positioned above the cylinders and the duration that each animal remained immobile as well as the latency to the first immobilization was measured over a 6-minute test period.

#### Novel object test

This is a free exploration paradigm providing the opportunity for the mice to explore a novel object in a non-threatening and familiar environment. For this test mice were first familiarized with the open field (50×50 cm×40 cm high) apparatus. Five days later, they were allowed to freely explore the open field in the absence of the object for 30 minutes (session 1, S1). Then, a novel object (a cup measuring 18 cm in height and 7 cm in diameter) was placed into the center of the open field. Mice were tested for an additional 30 minutes with the cup (Session 2; S2). The computer defined grid lines that divided the open field into five separate regions: one circular region in the center with a diameter of 20 cm and a surrounding region that was divided into quarters with gridlines that extended from the middle of each wall to the edge of the center region. The ratio S2/S1 measuring the distance and the time spent respectively in the presence (Session 2; S2) and in the absence (Session 1; S1) of the novel object were assessed by an automated video-tracking system.

#### Locomotor activity

Horizontal and vertical locomotor activities were measured in sixteen rectangular boxes by beam breaks via a fully computerized multi-box infrared sensitive motion-detection system. One mouse was placed in each box; sixteen mice were tested simultaneously. The apparatus consisted of sixteen rectangular boxes (length: 20.5 cm; width: 10.5 cm; high: 17.5 cm) isolated from one another by sound proof compartments. Two pairs of sending–receiving photoelectric cells were placed on each side of the activity boxes.

#### Water maze (WM)

Mice were required to locate a fixed hidden platform using distal extra-maze cues. On each training day, mice were released into the water facing the wall of the pool. Animals received three trials a day with a 5-minute inter-trial interval and at each trial the start position was changed. This procedure requires the hippocampus since the animal has to learn the positional relations among multiple independent environmental cues (“spatial relational memory”) in order to find the hidden platform. Daily trials lasted 60 seconds each and were stopped if the mouse reached the submerged platform where they were maintained for 15 seconds. If the platform was not found within 60 seconds mice were put on the platform and maintained there for 15 seconds. The probe test was performed by removing the platform and allowing each mouse to swim freely for 60 seconds inside the pool. The time that each mouse spent and the distance it swam in the target quadrant (where the platform was located during training) were recorded with a computerized video system. The water maze consisted of a circular pool (150 cm in diameter) filled with water mixed with a non-toxic white cosmetic adjuvant to obscure the platform and maintained at a temperature 23±2°C. The escape platform (15 cm in diameter) was submerged 1.0 cm below the surface. The maze was operationally sectioned into four equal quadrants of NW, NE, SW, and SE. Location of the platform remained in the centre of northwest quadrant throughout the training period. Differential visual spatial cues were placed on the walls surrounding the cylindrical tank and corresponding to quadrant corners. The swimming path of the animal was analyzed using a computerized video tracking system which calculated the latency to reach the platform, the length of the swim path and swim speed. WM experiment consisted of 10 days of training and a probe trial on day 11.

### Statistics

The normality of the data distribution was verified using the Shapiro-Wilk normality test and for homogeneity of variance we used Levene and Brown & Forsythe's tests. Data obtained for all control and mutant mice did satisfy the assumptions of normality, required for parametric analysis. Consequently, parametric statistics were performed. Statistical significance was assessed by ANOVA for repeated measures followed by post hoc comparisons (Newman-Keuls test) or by Student's t-test when appropriate. All values were expressed as mean ± s.e.m. Statistical significance was expressed as * = *P*<0.05; ** = *P*<0.01; *** = *P*<0.001.

## Supporting Information

Figure S1 Cellular characterization of cells expressing EGFP and ΔGR proteins in the cortex of *Eno2*-ΔGR/EGFP bigenic mice. Confocal illustrations of neurons from the cortex co-expressing EGFP protein and specific neuronal markers visualized with Cy^3^-conjugated antibodies. Distribution of EGFP and endogenous neuronal markers (NeuN, NSE, Glutamate) and merges of the two signals are shown.
(7.20 MB TIF)

Figure S2 Quantification of the EGFP-TetO-ΔGR transgene copy number by real time quantitative PCR (qPCR). The quantification was performed by relating the PCR signal to a standard curve. *Eno2*-ΔGR/EGFP bigenic mice contained 2 copies of the EGFP-TetO-ΔGR expression vector.
(0.36 MB TIF)

Figure S3 Comparison of the brain expression pattern of two independent *Eno2*-ΔGR/EGFP bigenic lines. The DG positive line (L27) used in our experiments had a restricted expression pattern in the DG and few positive cells in the cortex, whilst the DG negative line (L23) expressed the ΔGR in the dorsal and ventral striatum, in the cortex, in the CA1 of the hippocampus but not in the DG.
(9.53 MB TIF)

Figure S4 Behavioral comparisons between the DG positive line (L27) and the DG negative line (L23). Any of the behavioral phenotypes that were significantly modified in Line 27 showed significant changes in Line 23. Elevated Plus Maze test; (A-A') Time in open arms (OA)/total time (%) (t_20_ = −0.623 p>0.538 for A'), (B-B') Time in open arms (sec) (t_20_ = −0.968 p>0.343 for B'), (C-C') Entries in open arms extremities (Nb) (t_20_ = −1.473 p>0.155 for C'), (D-D') Closed arms entries (Nb) (t_20_ = −0.223 p>0.824 for D'). Emergence test; (E-E') Latency to exit (sec) (t_20_ = −0.210 p>0.835 for E'), (F-F') Locomotor activity (m) (t_20_ = −0.188 p>0.851 for F'). Forced swim test; (G-G') First immobilization latency (sec) (t_22_ = −0.078 p>0.937 for G'), (H-H') Duration of immobility (sec) (t_22_ = −0.450 p>0.655 for H'). Statistical measures for panels A-H are given within the Results section. Values shown are means +/− sem. * = *P*<0.05; ** = *P*<0.01.
(4.46 MB TIF)
