# Supplementary figures and images for: Transcriptional Effects of Glucocorticoid Receptors in the Dentate Gyrus Increase Anxiety-Related Behaviors
Source: PLoS One. 2009 Nov 2;4(11):e7704. doi: 10.1371/journal.pone.0007704 (PMC2765620; doi:10.1371/journal.pone.0007704)

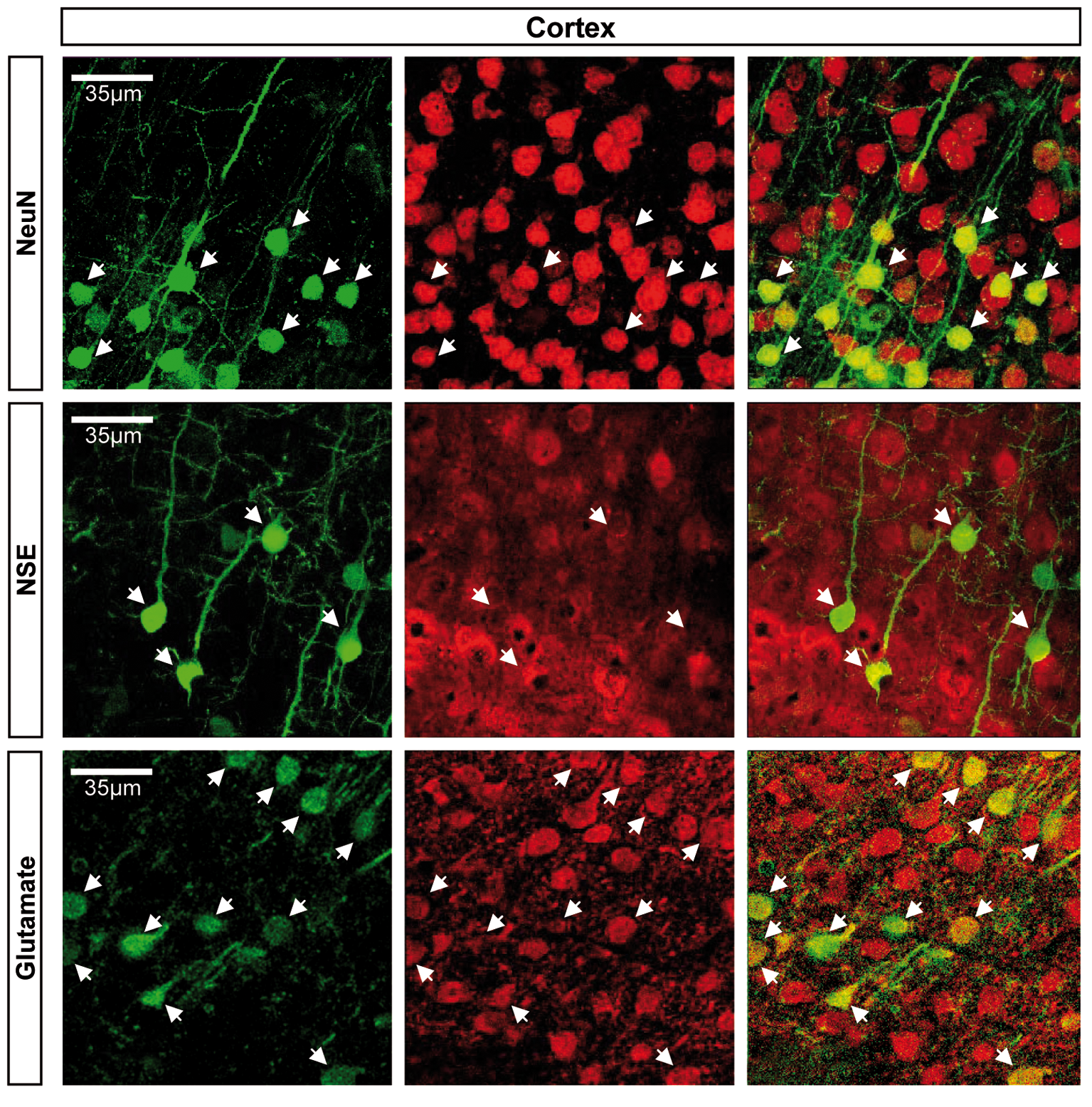

Supplement: Figure S1 — Cellular characterization of cells expressing EGFP and ΔGR proteins in the cortex of Eno2-ΔGR/EGFP bigenic mice. Confocal illustrations of neurons from the cortex co-expressing EGFP protein and specific neuronal markers visualized with Cy3-conjugated antibodies. Distribution of EGFP and endogenous neuronal markers (NeuN, NSE, Glutamate) and merges of the two signals are shown. (7.20 MB TIF) [file pone.0007704.s001.tif]

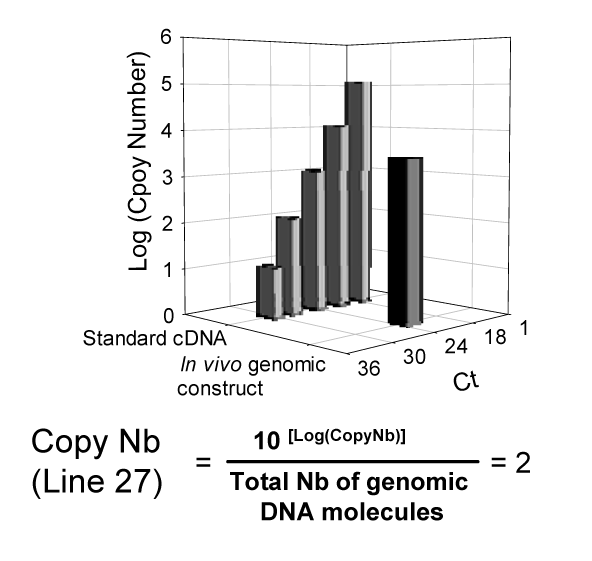

Supplement: Figure S2 — Quantification of the EGFP-TetO-ΔGR transgene copy number by real time quantitative PCR (qPCR). The quantification was performed by relating the PCR signal to a standard curve. Eno2-ΔGR/EGFP bigenic mice contained 2 copies of the EGFP-TetO-ΔGR expression vector. (0.36 MB TIF) [file pone.0007704.s002.tif]

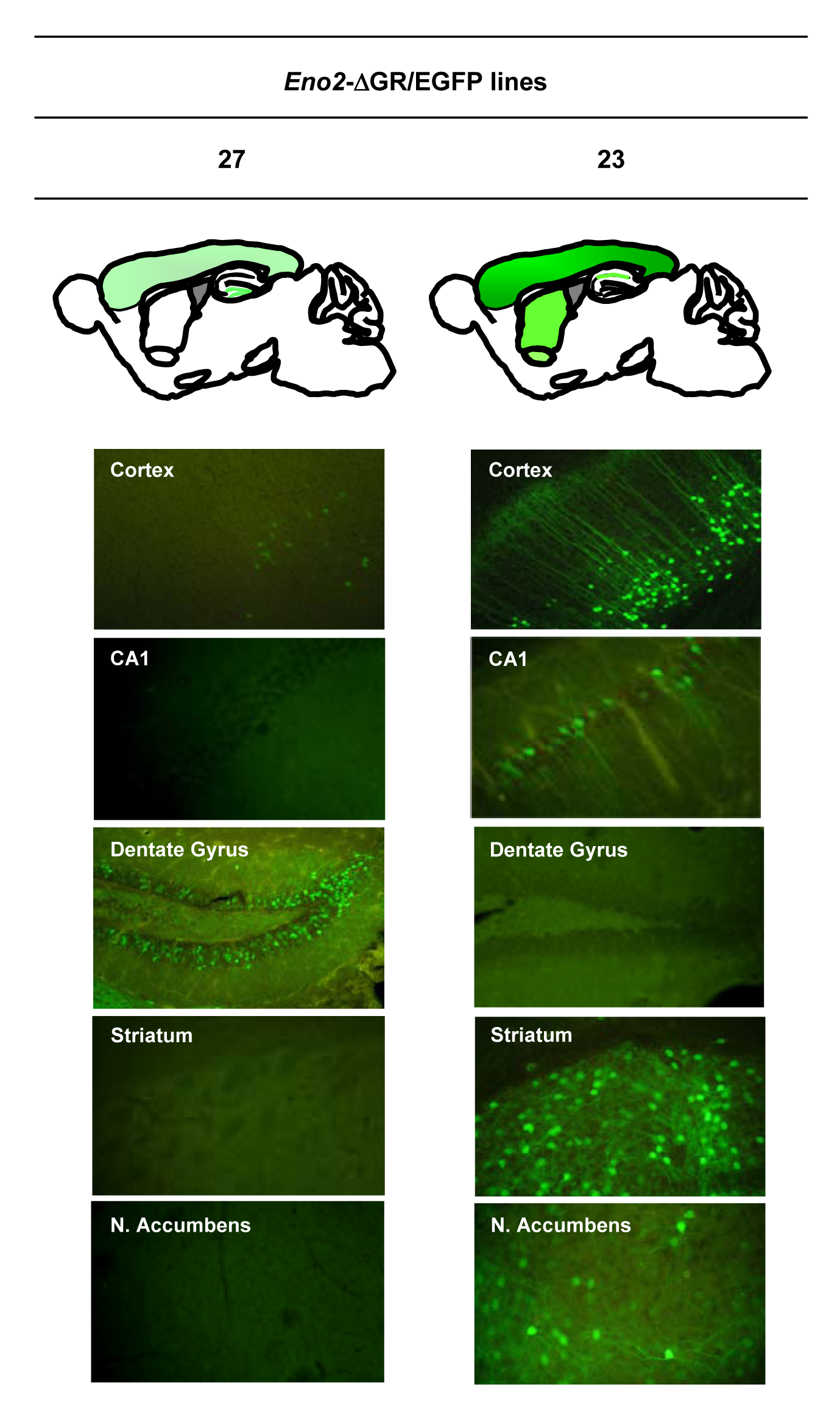

Supplement: Figure S3 — Comparison of the brain expression pattern of two independent Eno2-ΔGR/EGFP bigenic lines. The DG positive line (L27) used in our experiments had a restricted expression pattern in the DG and few positive cells in the cortex, whilst the DG negative line (L23) expressed the ΔGR in the dorsal and ventral striatum, in the cortex, in the CA1 of the hippocampus but not in the DG. (9.53 MB TIF) [file pone.0007704.s003.tif]

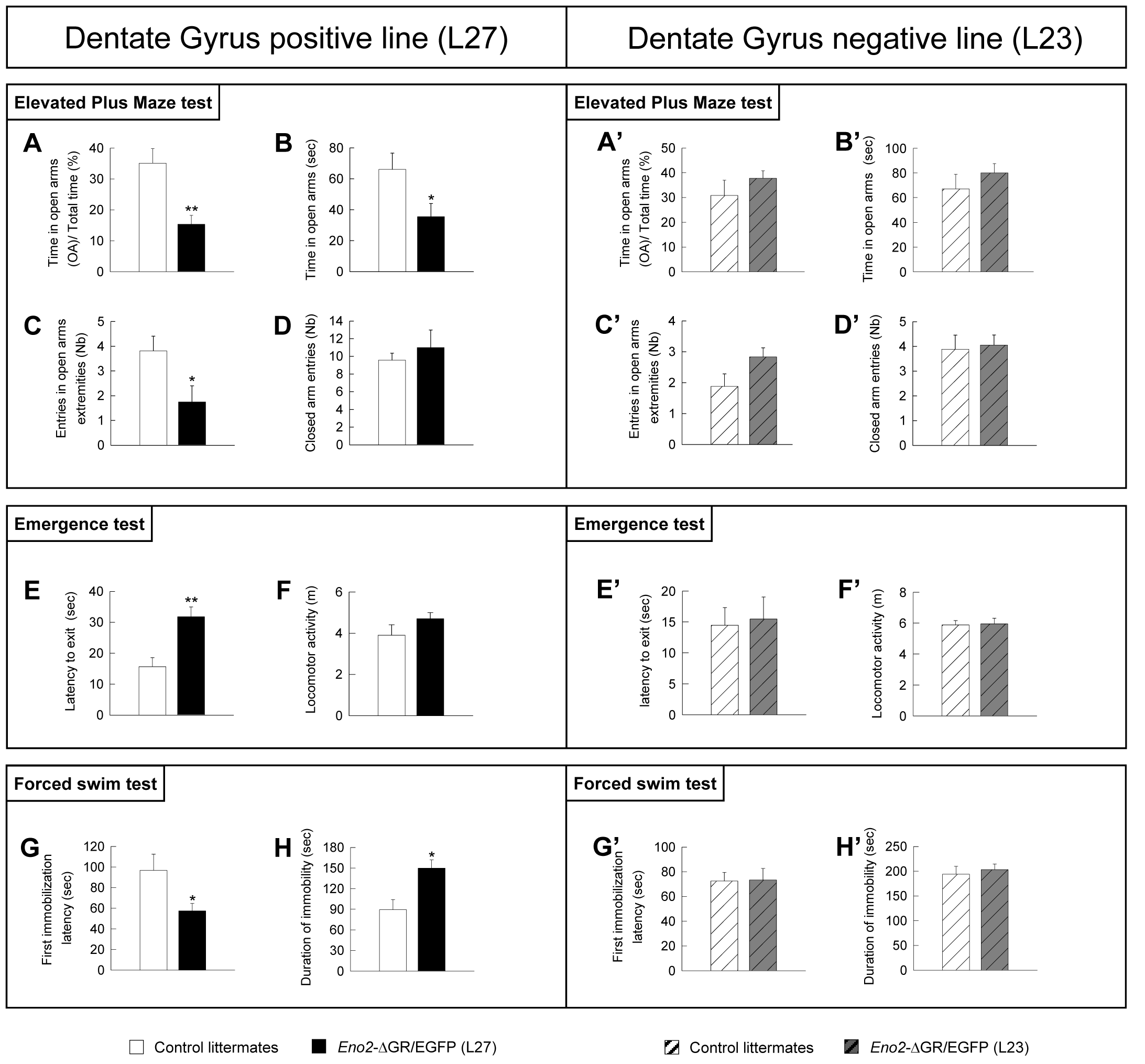

Supplement: Figure S4 — Behavioral comparisons between the DG positive line (L27) and the DG negative line (L23). Any of the behavioral phenotypes that were significantly modified in Line 27 showed significant changes in Line 23. Elevated Plus Maze test; (A-A') Time in open arms (OA)/total time (%) (t20 = −0.623 p>0.538 for A'), (B-B') Time in open arms (sec) (t20 = −0.968 p>0.343 for B'), (C-C') Entries in open arms extremities (Nb) (t20 = −1.473 p>0.155 for C'), (D-D') Closed arms entries (Nb) (t20 = −0.223 p>0.824 for D'). Emergence test; (E-E') Latency to exit (sec) (t20 = −0.210 p>0.835 for E'), (F-F') Locomotor activity (m) (t20 = −0.188 p>0.851 for F'). Forced swim test; (G-G') First immobilization latency (sec) (t22 = −0.078 p>0.937 for G'), (H-H') Duration of immobility (sec) (t22 = −0.450 p>0.655 for H'). Statistical measures for panels A-H are given within the Results section. Values shown are means +/− sem. * = P<0.05; ** = P<0.01. (4.46 MB TIF) [file pone.0007704.s004.tif]
